# Development of wearable multi-lead ECG measurement device using cubic flocked electrode

**DOI:** 10.1038/s41598-022-24043-6

**Published:** 2022-11-11

**Authors:** Toshihiro Takeshita, Manabu Yoshida, Yusuke Takei, Atsushi Ouchi, Akinari Hinoki, Hiroo Uchida, Takeshi Kobayashi

**Affiliations:** 1 Sensing System Research Center, National Institute of Advanced Industrial Science and Technology (AIST), Tsukuba, 305-8564 Japan; 2 Graduate School of Medicine, Nagoya University, Nagoya, 466−8550 Japan

**Keywords:** Cardiology, Diseases, Health care, Engineering, Materials science

## Abstract

This paper describes the fabrication and fundamental evaluation of the cubic flocked electrode (CFE), which is a dry electrode that is fabricated using electrostatic flocking technology. The development of a wearable multi-lead electrocardiogram (ECG) measurement device using the CFE is also reported. To enable the measurement of ECG signals with sufficient quality for medical applications, the occurrence of motion artifacts (MAs) is the most important problem to be overcome. Therefore, it is necessary to stabilize the contact between the patient’s skin and the dry electrode. Because the CFE developed in this work offers both contact stability and flexibility, it is expected to enable ECG measurements with low MA levels. In this study, it is demonstrated that the number of MAs caused by respiration can be reduced when the CFE contact is made at a contact pressure of approximately 500 Pa using MA evaluation equipment that was developed in-house. Additionally, a wearable multi-lead ECG is designed and fabricated based on this contact pressure (500 Pa). The results of the demonstration experiment show that the ECG measurements are successful to the same extent as a conventional medical device.

## Introduction

Vital sign monitoring using wearable sensors enables health management in daily life. For example, various wearable sensors have been reported for pulse rate^1–3^, peripheral oxygen saturation (SpO_2_)^4,5^, body temperature^6–8^, blood flow^9,10^, and electromyography measurements^11,12^. In particular, the demand for wearable measurement sensors for electrocardiogram (ECG) measurements^13–17^ is high because heart disease is among the most prevalent causes of death worldwide^18^. For example, the Asahi Kasei Zoll Medical Corporation is selling the “Life Vest”, which integrates an ECG measurement function using dry electrodes and an electric shock function into the vest^19^. When ventricular fibrillation occurs during sleep, the Life Vest can save the patient's life by detecting the ventricular fibrillation and automatically administering an electric shock. As described above, wearable ECG measurement devices are widely used in health management applications and medical applications.

To measure ECG signals for medical applications such as 12-lead ECG, Holter ECG monitoring, and Mason-Likar (ML) ECG, electrodes must be placed on the patient’s chest and torso. Therefore, a clothing-type wearable device is suitable for use in medical applications. In addition, from the viewpoints of wearability and hygiene, the electrode should be a dry type rather than a wet type^20–23^. However, one of the most important problems to be overcome when performing ECG measurements with dry electrodes is motion artifacts (MAs). MAs are fluctuations of the ECG signal due to body movement. Because gels are not used with dry electrodes, the mechanical contact between the skin and the electrode is unstable. Therefore, even minor movements such as breathing can cause changes in the impedance and the resting potential between the skin and the electrodes. As a result, these electrical changes distort the baseline of the ECG. One way to reduce MAs is to increase the contact pressure acting between the skin and the electrodes. Inoue’s group reported stable ECG measurements where they improved the contact pressure by impregnating the sponge with conductive ink^24^. In addition, Cardon’s group reported dry electrodes formed into the shape of an array of pillars that improve the local contact pressure^25^. However, MAs and patient discomfort are in a trade-off relationship. Therefore, contact pressure optimization is important.

The author’s group has been developing a dry electrode using electrostatic flocking technology^26,27^ and wearable multi-lead ECG measurement devices^28^. However, in previous reports, we improved the contact pressure by wrapping the wearer’s chest with a corset over the compression wear. This method was very uncomfortable for the wearer. Therefore, in this study, we have developed a novel dry electrode called the “cubic flocked electrode (CFE)” that can improve the contact pressure locally without the need for a corset. The CFE, in which the surface of a urethane form is electrostatically flocked with Ag-plated fibers, is a dry electrode that offers contact stability and high flexibility. Therefore, high MA resistance can be expected when using the CFE under appropriate contact pressure.

In this paper, we first describe the CFE fabrication method and the fundamental characteristics of this electrode. It is shown that the dry electrode material can be applied uniformly to a three-dimensional urethane form surface using electrostatic flocking technology. Next, to estimate the contact pressure value that can enable ECG measurements to be performed without MAs, an MA reproduction and evaluation experiment was conducted using a skin phantom. In this experiment, we aimed to prevent MAs from occurring because of body movements caused by normal respiration. Based on the contact pressure value obtained from the MA reproduction experiment, we designed a wearable multi-lead ECG measurement device using the CFE. This wearable multi-lead ECG measurement device was developed for measuring 12-lead ECG at home without a doctor. Since the measurement is assumed to take several minutes in a resting state, motion artifacts due to normal respiration are dominant. Therefore, we designed the device so that the contact pressure can reduce motion artifacts caused by normal respiration.

## Results

### Cubic flocked electrode

**Figure 1:**
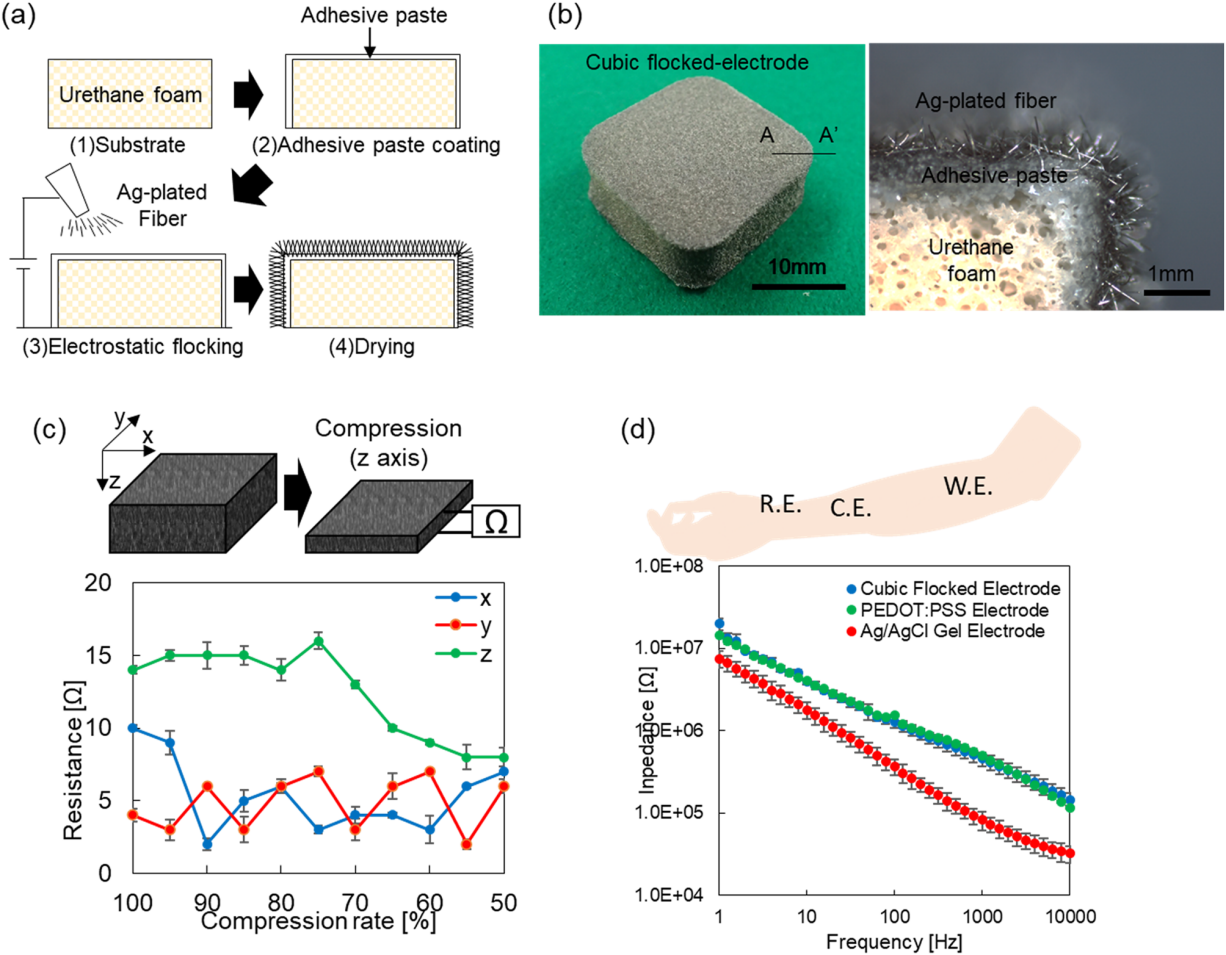
Picture and fundamental evaluation of the cubic flocked electrode fabricated in this work. (**a**) Image of CFE fabrication process using electrostatic flocking technology. (**b**) Photograph of the CFE and cross-sectional view (at the A-A’ line). (**c**) Resistance changes in the CFE when compressed in the directions of the *x*, *y*, and *z* axes. (**d**) Impedance spectra of the CFE, PEDOT:PSS electrode, and Ag/AgCl gel electrode, measured in the 1 Hz to 10 kHz frequency range when in contact with skin (forearm) at 200 Pa. The spectra show the mean values and standard errors (measurements were taken five times).

Figure 1a shows the CFE fabrication process using electrostatic flocking technology, where Ag-plated fibers were formed on the surface of a urethane form. The fibers were charged at 50 kV. Therefore, the fibers stand vertically against the urethane form surface, as shown in Fig. 1b. The CFE has dimensions of 20 mm × 20 mm and is 12 mm thick. The hardness of the CFE is 20° (SRIS 0101/Asker C). The flocking process is described in detail in the “Methods” section.

First, we evaluated the fundamental characteristics of the CFE. Because the CFE is flexible, it can be deformed greatly by compression. The effect of this deformation on the resistance value of the CFE itself was therefore evaluated. Figure 1c shows the changes in the resistance value when the CFE is compressed in the three axial (*x*, *y*, and *z*) directions. The compression rate ranged from 10 to 50%. The resistance value fluctuation is the largest along the z-axis (from 14.1 Ω at 100% to 8.0 Ω at 50%). The resistance fluctuations along the *x* and *y* axes were smaller than those along the *z-axis* (less than 10 Ω). However, when compared with the skin-electrode impedance, which is approximately a few megaohms, the resistance change was small enough to be negligible. Therefore, large-scale deformation of the CFE itself does not affect the ECG measurements.

Figure 1d shows the skin-electrode impedance characteristics of the CFE, a poly(3,4-ethylene dioxythiophene) polystyrene sulfonate (PEDOT: PSS) electrode (C3fit IN-pulse, Goldwin Inc., Japan), and an Ag/AgCl electrode (Red Dot, 3M, USA) with gel. The measurement frequency range for the impedance is from 1 Hz to 10 kHz and the measurement position was at the forearm, as shown in the figure^29^. Because the impedance is dependent on the contact pressure^28^, the contact pressure between the skin and the electrode was fixed at 200 Pa. From this result, the impedance of the CFE was higher than that of the Ag/AgCl electrode with gel. However, when compared with the PEDOT: PSS electrode, which is an industrial product, the impedance was at the same level for both electrodes. This result shows that the impedance is small enough to allow the CFE to be applied to ECG measurements.

### MA reproduction experiment

To evaluate the MA durability of the CFE, an MA reproduction experiment was conducted using a skin phantom. The purpose of this experiment is also to obtain the appropriate contact pressure between the skin and the CFE for the design of the wearable multi-lead ECG measurement design. The magnitude of the motion artifact *V*_*ma*_ is given by the following equation^30^.

1 $${\mathrm{V}}_{\mathrm{ma}}=2\left[\Delta {\mathrm{V}}_{\mathrm{dc}-\mathrm{offset}}+\Delta {\mathrm{Z}}_{\mathrm{skin}-\mathrm{electrode}} \left(\frac{\left(\frac{{\mathrm{V}}_{\mathrm{ECG}}}{2}\right)+{\mathrm{V}}_{\mathrm{dc}-\mathrm{offset}}}{{\mathrm{Z}}_{\mathrm{in}}}+{\mathrm{I}}_{\mathrm{bias}}\right)\right]$$

*V*_*dc-offset*_, *Z*_*skin-electrode*_, *V*_*ecg*_, and *I*_*bias*_ are the offset voltage generated at the skin-electrode interface, the impedance between the skin and the electrode, the ECG signal voltage, and the input bias current of the amplifier, respectively. Δ*V*_*dc-offset*_ and Δ*Z*_*skin-electrode*_ are major causes of MAs. The purpose of this paper is to reduce both Δ*V*_*dc-offset*_ and Δ*Z*_*skin-electrode*_, and to show experimentally the type of pressure and conditions under which *V*_*ma*_ can be reduced by using a raised electrode.

**Figure 2:**
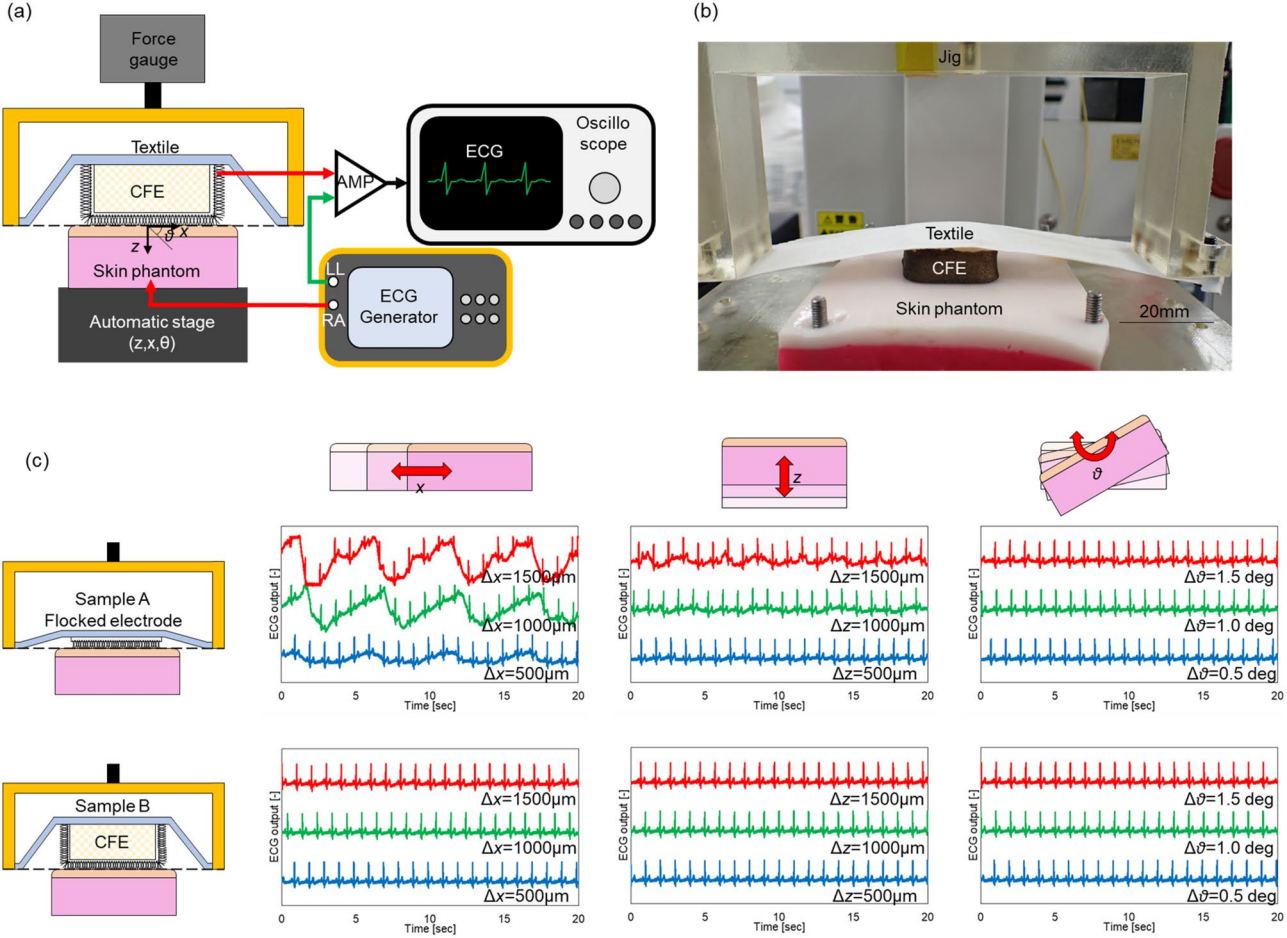
MA evaluation when measuring ECG using a skin phantom. (**a**) Experimental setup of MA evaluation equipment, and (**b**) photograph of contact part. (**c**) Experimental results of the MA evaluation experiments when the automatic stage displaced the *x*-axis (Δ*x* = 500 µm, 1000 µm, 1500 µm), the *z*-axis (Δ*z* = 500 µm, 1000 µm, 1500 µm), and the *θ*-axis (Δ*θ* = 0.5°, 1.0°, 1.5°). Sample A is the textile on which the flocked electrode was formed directly. Sample B is the CFE, which was attached to the textile.

Figure 2a,b show the experimental system. The skin potential of the right arm (RA) position, which was generated using an ECG simulator (ProSim8, Fluke Corp., USA), was connected to the skin phantom (HXBNXTB858510MX, Wet Lab Inc., Japan). The CFE was attached to the surface of the skin phantom and was then connected to an ECG amplifier (Analog Devices, USA). The CFE was glued to a textile (MCM3749, Under Armour, USA), which is the same material that is used for the wearable multi-lead ECG measurement device described later in the paper. The edge of the textile was fixed to a jig. The contact pressure between the electrode and the skin phantom was measured using a force gauge. Additionally, the skin potential of the left leg (LL) was connected to the amplifier directly. The ECG signal was recorded using an oscilloscope as the output ECG signal. The skin phantom was attached to an *x*, *z*, and *θ* axial automatic stage. This automatic stage moved in a round trip such that displacement occurred between the electrodes and the skin phantom. As a result, an MA was reproduced. Note that the center of the axis of rotation of *θ* is the contact point between the skin phantom and the electrode.

Two samples were prepared to compare their ECG signal quality levels. Sample A is a flocked electrode that was formed on the textile directly. Sample B is the CFE attached to the textile, as shown in Fig. 2c. These samples were placed in contact with the skin phantom such that the fixed part of the cloth was aligned with the surface of the skin phantom. This contact state is a reproduction of the contact state of the actual wearable device itself. The contact pressures measured using the flocked electrode sample and the CFE sample were 57.3 Pa and 495.2 Pa, respectively, as calculated using the output of a force gauge. The ECG signal was measured when the automatic stage reciprocated on the *x*-axis (Δ*x* = 500 µm, 1000 µm, 1500 µm), the *z*-axis (Δ*z* = 500 µm, 1000 µm, 1500 µm), and the *θ*-axis (Δ*θ* = 0.5°, 1.0°, 1.5°). These displacement values were calculated based on the chest expansion rate during respiration^31^. The frequency of the stage motion was 14 round trips/min, which was equivalent to the average human breathing frequency. The measurement time was 20 s.

About the results for Sample A, the largest MA was observed when the stage moved along the *x*-axis. The MA is confirmed, even at Δ*x* = 500 μm. This is because of the occurrence of slippage. Because the initial contact pressure was 57.3 Pa, the flocked electrode could not follow the *x*-axis displacement, and the skin phantom-electrode interface slipped as a result. For the *z*-axis and the *θ*-axis, the MA was suppressed because the textile absorbed the displacement, which meant that the MA was smaller than the *x*-axis displacement. In contrast, in the Sample B results, MAs were hardly observed on the *x*-axis, the *z*-axis, and the *θ*-axis. It is believed that this result was caused by the increased contact pressure when making the cubic shape of the urethane form. This means that the slippage was suppressed by the shear deformation of the urethane form. The results above show that the occurrence of MAs can be reduced by using the CFE. Furthermore, we obtained a design indicator that contact pressure of 500 Pa or more can reduce the number of MAs being caused by respiration.

### Wearable multi-lead ECG measurement device

**Figure 3:**
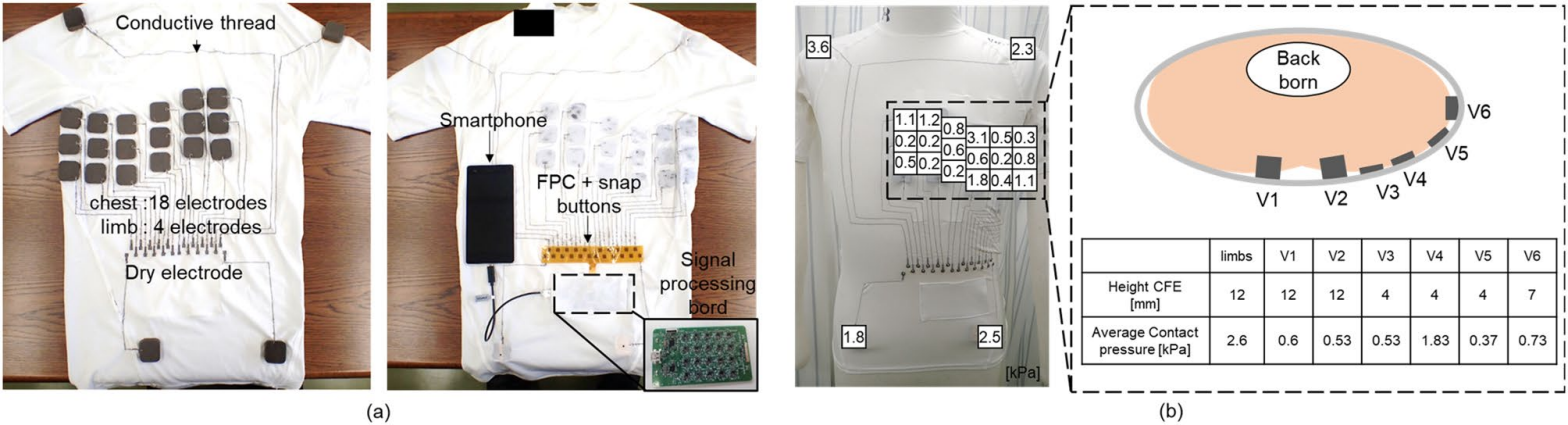
Multi-lead ECG wearable measurement device. Photographs of (**a**) rear side, and (**b**) front side of the wearable device. (**c**) Contact pressure distribution on the CFE, which was attached between the wearable device and the torso.

Figure 3a shows the wearable multi-lead ECG measurement device that we developed in this work. The view is from the rear side of the device. Compression wear (MCM3749, Under Armour) was used as a substrate textile to increase the contact pressure between the skin and the CFE. On the compression wear surface, 18 CFEs were attached around the chest area, and four CFEs were attached to the limbs using adhesive paste. The positions of these electrodes were designed to measure the V1–V6 signals of the Mason-Likar (ML) lead ECG signals, which are equivalent to the V1–V6 signals of the 12-lead ECG signals^32^. The 18 electrodes located around the chest can be divided into six upper electrodes, six middle electrodes, and six lower electrodes. The reason for this division procedure is to reduce the influence of electrode positioning errors due to differences in the way that individual patients wear the device. These electrodes were connected to the pads in the stomach position using conductive thread (AGposs, Mitsufuji Corp., Japan) and were sewn in position using a sewing machine. The pads were also fabricated using the electrostatic flocking technology and were connected to corresponding pads on the front side by through-wiring. The conductive threads were protected and covered with a film to prevent the threads from attaching to the patient’s skin directly because direct attachment can cause noise on the ECG signals. The measurement system is described in detail in the “Methods” section. Figure 3b shows the evaluation of the contact pressure between the CFE surface and the skin of a torso (CPLM, TOMANE CO., LTD., Japan). The contact pressure was measured by the insertion of a balloon that was connected to an air pressure measurement system (AMI3037-2, AMI Tec Inc., JAPAN). To optimize the contact pressure according to the MA evaluation experiment, the CFE’s height was designed by checking several contact pressures in order up to more than 500 Pa. Finally, the heights of the CFEs were determined as shown in Fig. 3b. The average contact pressures with the optimized CFE at positions V1–V6 were 0.6 kPa, 0.53 kPa, 0.53 kPa, 1.83 kPa, 0.37 kPa, and 0.73 kPa, respectively. These results show that the contact pressures are almost all more than 500 Pa, which was indicated in the MA evaluation results.

**Figure 4:**
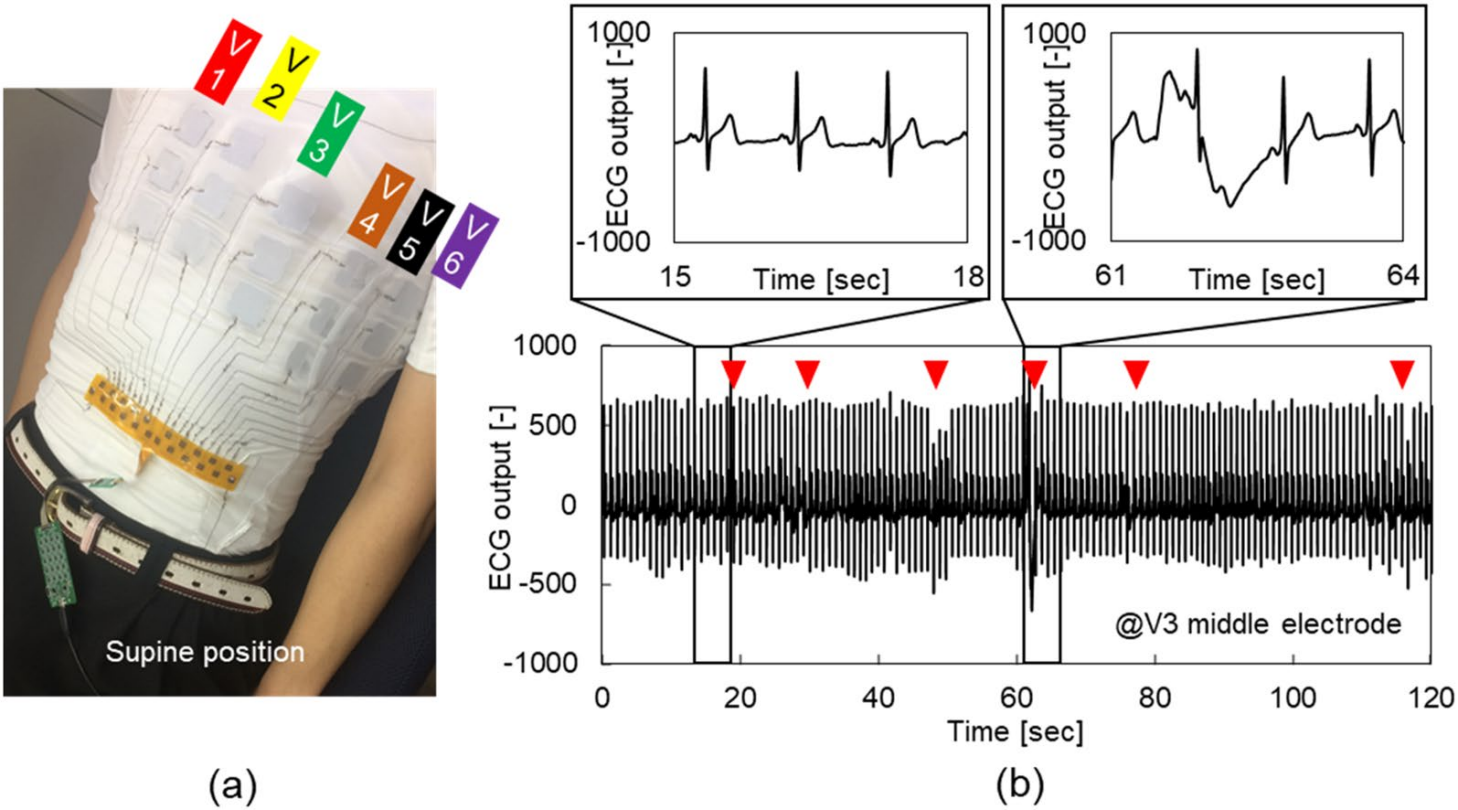
Experimental results of multi-lead ECG measurements. (**a**) Photograph of the experiment. The subject put on the wearable device and rested in the supine position while breathing freely. (**b**) ECG signal (from V3 position of middle electrodes) from a 2 min measurement period.

Figure 4a shows a photograph of the ECG measurement experiment with the wearable multi-lead ECG measurement device. The experimental protocol was as follows.

1. Put on the wearable device without gel.
2. Measure the multi-lead ECG signal in the spine position while breathing freely (2 min).
3. Take off the wearable device.
4. Measure the 12-lead ECG signal using a medical product (Smart ECG, ECG Lab Corp., Japan) in the spine position while breathing freely (2 min). The electrodes were placed by a medical doctor. The number of subjects for this experiment was 10.

Figure 4b shows the ECG signals acquired in 2 min at the V3 position of the middle six electrodes. Both signals with MAs and signals without MAs can be observed from the ECG signals of this 2 min period. In a signal without MAs, the P, Q, R, S, and T waveforms can be observed clearly. In contrast, the signal with the largest MA in this experiment was detected as shown in the upper right area of this figure. From this signal, while the R waveform is the exception, the P, Q, S, and T waveforms could not be observed because of the fluctuations in the baseline. However, these fluctuations occurred only six times in 2 min (marked by red triangles). Furthermore, the fluctuations occurred for only a few seconds and soon disappeared. This result indicates that the cause of the baseline fluctuation was a slight movement of the human body rather than breathing.

**Figure 5:**
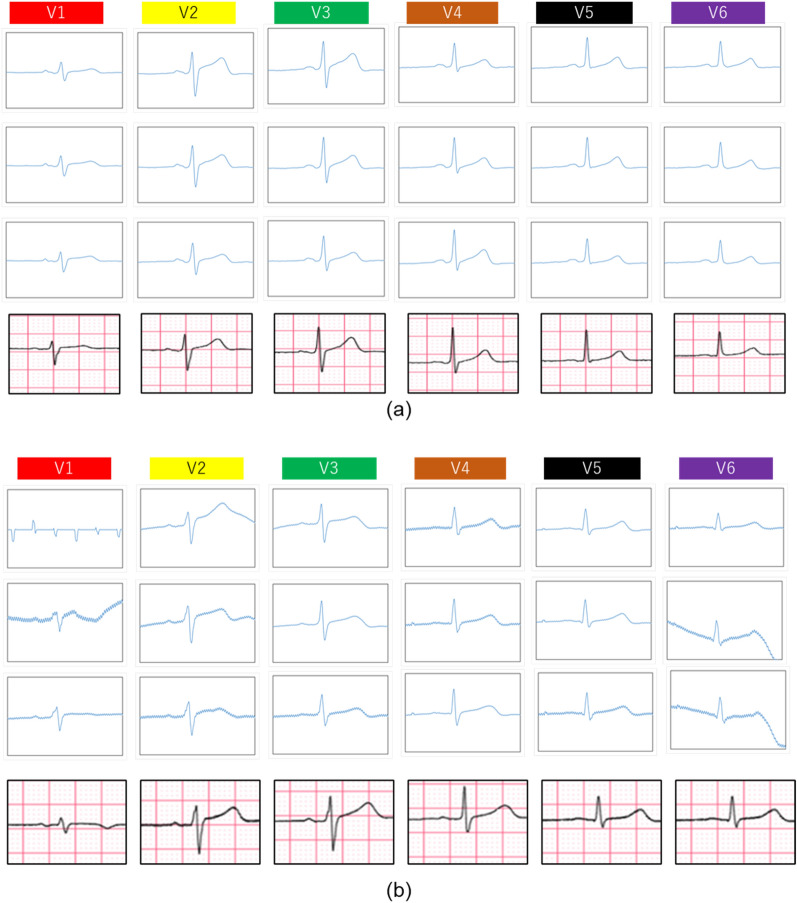
The 18-lead ECG signals measured by the wearable multi-lead ECG measurement device and by a commercial medical product around the chest. (**a**) Best quality ECG from 10 subjects, and (**b**) worst quality ECG from 10 subjects.

Figure 5 shows the ECG signals measured by the wearable multi-lead ECG measurement device (18 electrodes) and by medical products (six electrodes). Figure 5a shows the ECG waveform of a subject who succeeded in producing a stable measurement. About the 18 lead ECG signals, we successfully acquired waveforms of sufficiently good quality to allow the P, Q, R, S, and T waves to be identified in all leads. In addition, the ECG signal waveforms for each lead are similar to those of the six-lead ECG signal obtained using the medical product. This confirms that the proposed ECG offers the same quality as the medical product. In contrast, Fig. 5b shows the ECG waveform for a subject whose measurements were not stable. Fluctuations of the baseline and noise are observed in signals V1, V2, and V6. Signal V1 of the upper electrode cannot be confirmed, even with the R waveform. In contrast, in signals V3 to V5, we have achieved stable ECG signal measurements to some extent. Because the subject was slender (chest circumference: 86.0 cm) when compared with the other subjects (average chest circumference: 92.2 cm), it is considered that stable ECG measurements are possible, even in the V1, V2, and V6 signal areas, by designing wearable multi-lead ECG devices to be a specific size. The results of the demonstration experiment above show that it is possible to measure an ECG at the same level as a medical product with the patient in a resting state by using the proposed wearable multi-lead ECG measurement device with the CFE.

## Discussion

In this paper, the fundamental characteristic evaluation of the CFE, the quantitative evaluation of the MAs, the compression design for the wearable multi-lead ECG measurement device using the CFE, and the results of demonstration tests performed in a hospital are described.

About the internal resistance fluctuations that occurred when the CFE was greatly deformed, the resistance change was several ohms, even at a compression rate of 50% in *x*, *y*, and *z* axial compression. As mentioned in a previous report^28^, the electrostatic flocking-based electrode has characteristics of both high elasticity and high conductivity. Therefore, the change in resistance of the CFE can be reduced even under high compression rate conditions.

Next, about the results of the MA reproduction experiment, ECG measurements were performed when the skin phantom and the electrode were displaced on the *x*, *z*, and *θ* axes. In particular, the influence of the *x*-axial displacement was the largest among the displacements on these three axes. As mentioned earlier, this displacement is due to a slip at the skin phantom-electrode interface. This slip is dependent on the initial contact pressure and the length of the silver-plated fiber. During small movements of the body such as breathing, MAs did not occur in the sample when using the CFE. However, MAs can occur during larger movements such as walking and exercise. However, increasing the contact pressure is not the preferred solution in terms of both wearability and patient comfort. Therefore, it is important to design a nonslip dry electrode. In future work, we will investigate the relationship between the length of the silver-plated fibers and the slippage, and the MAs caused by this slippage.

Next, the ECG measurement results for two subjects from a total of 10 subjects are also presented (Fig. 5). About the results of the one subject who succeeded in producing stable ECG measurements, it is probable that all electrodes were in stable contact with the subject because the size of the wearable multi-lead ECG measurement device was fitted well. These results were also in good agreement with those from a medical product for 18-lead ECG. Generally, in a 12-lead ECG, the peak of the R wave decreases when the electrode position on the chest rises. Additionally, the peak of the R wave increases when the electrode position on the chest falls^33^. This tendency was also confirmed in the experiments in this work. These results confirmed that we successfully measured the ECG, with results that are consistent with conventional medical findings.

In contrast, about the results of the subject who did not achieve stable ECG measurements, the cause of the instability is the size mismatch that was described above. In particular, the ECG was unstable in the epigastrium (V1, V2) and the axilla (V6). It is believed that this instability was not due to the electrode height of the CFE but occurred because the pressing force of the clothing itself that was holding the electrode was weak. The actual height of the CFE was 12 mm at the epigastrium and 7 mm at the armpit (and 4 mm for V3 to V5). The contact was probably unstable despite these electrode heights because the pressing force of the clothing acting on the back of the CFE was low. This result illustrates that it is important to design the CFE and the wearable device to suit the wearer's body shape.

Finally, we discuss the specification comparisons between different dry electrodes for 12-lead ECG measurement as shown in Table 1. The novelty of the proposed electrode is that stable ECG can be measured with low contact pressure. Conventional dry electrodes need a contact pressure of approximately 2.0 kPa or more to stably measure ECG. Also, many papers do not mention contact pressure. Even with non-contact dry electrodes, there is a cloth between the electrode and the skin, so the contact pressure between the cloth and the skin is an important factor. On the other hand, with CFE, stable ECGs can be measured at 0.5 kPa in skin phantom experiments and 0.77 kPa (average) in demonstration tests. We believe that low contact pressure is an important characteristic and achievement of CFE because low contact pressure brings comfort to the wearer.

**Table 1:** The specification comparisons between different dry electrodes for 12-lead ECG measurement.

	Hsu et al.^34^	Anna et al.^35^	Inoue et al.^24^	Takeshita et al.^28^	Proposed
ECG	12-Lead ECG	12-Lead ECG	12-Lead ECG	12-Lead ECG	12-Lead ECG
Are of electrode (cm^2^)	6.16	16.0	4.0	1.0	4.0
Electrode material	Copper	Silber	Conductive ink (silver)	Ag-plated fiber	Ag-plated fiber
Activity in measurement	Sitting Walking	Sitting Lying Walking	Sitting Walking Jogging	Sitting Lying	Sitting Lying
Contact pressure	– (Non-contact electrode)	–	3.3–4.9 kPa	2.0 kPa	0.5–0.77 kPa

In summary, we have reported the development of a wearable multi-lead ECG measurement device using CFEs. In future work, we aim to realize a dry electrode that can perform stable ECG measurements without MAs not only during breathing but also while the patient is walking and taking exercise.

## Methods

### Fabrication of cubic flocked electrode

(1st step) The cubic urethane form was 20 mm × 20 mm in size and 10 mm thick. The hardness of the urethane form was 20° (SRIS 0101/Asker C). (2nd step) Adhesive paste (UNIBINDER NU-84, Co., Ltd., Japan) was sprayed around the urethane form. (3rd step) Subsequently, Ag-plated fibers (AGposs cut fiber 1.7T, 0.5 mm; Mitsufuji Corporation, Japan) were formed around the urethane form using electrostatic flocking technology. The fibers were 500 µm long and have a diameter of 18 µm. The fibers were charged up to 50 kV and then sprayed on the urethane form. Therefore, the fibers stand vertically against the surface of the urethane form. (4th step) Finally, the urethane form was dried in a furnace at 100 °C for 30 min.

### Fabrication of wearable multi-lead ECG measurement device

**Figure 6:**
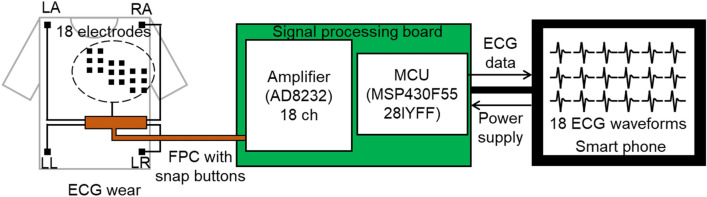
Wearable multi-lead ECG measurement device and data acquisition system.

The multi-lead ECG wear and measurement system is shown in Fig. 6. The signals measured by the CFE at each position were connected to the flexible printed circuit (FPC) via snap buttons on the wearable device. Therefore, the wearable device and the FPC can be attached and detached both mechanically and electrically. The FPC is connected to a signal processing board with amplifiers (AD8232, Analog Devices) and a microcontroller unit (MCU; MSP430F55281YFF, Texas Instruments, USA). Eighteen amplifiers are included to measure the V1–V6 signals at the upper six electrodes, the middle six electrodes, and the lower six electrodes. These signals are then transmitted to a smartphone or personal computer (PC) via wires and the signals can thus be measured. The smartphone or PC supplies the power to the signal processing board.

### Demonstration test

The authors declare that the Ag-plated fiber developed in this work does not have skin-irritating properties, as confirmed by the results of a primary skin irritation test (MTT assay). Additionally, the experimental protocols in this paper were approved by the IRB of Ethics in Applied Biomedical Engineering and Technology (71120030-A-20181002-001) in the National Institute of Advanced Industrial Science and Technology (AIST). Furthermore, the authors declare that all experiments were carried out while following the relevant guidelines and regulations. All volunteers provided informed signed consent to participate in the study. There were no volunteer complaints including skin itching or rashes in the demonstration test.

## Data Availability

The datasets generated during and/or analyzed during the current study are available from the corresponding author upon reasonable request.
